# Assessment of increased knowledge about traffic accidents prevention, one month after a presentation included in the program “it can happen to you” of AESLEME

**DOI:** 10.1038/s41393-023-00887-1

**Published:** 2023-03-24

**Authors:** Mar Cogollos-Paja, Juan Angel García-Reneses, Rafael Herruzo

**Affiliations:** 1 Psychologist, Director of AESLEME, Madrid, Spain; 2 Physician specialised in Rehabilitation, Vice-President of AESLEME, Madrid, Spain; 3 Professor of Preventive Medicine and Public Health, Universidad Autónoma de Madrid, Madrid, Spain

**Keywords:** Pathogenesis, Risk factors

## Abstract

**Abstract:**

Road traffic accidents are a real pandemic and incur expenses amounting to 1–2% of every country’s GDP. AESLEME (Association for the Study of Spinal Cord Injuries) has celebrated its 30th anniversary here in Spain. AESLEME’s instructors are health workers and people with spinal cord injuries caused by road accidents: their presentations—teaching road safety and sharing information on irreversible injuries—are enhanced by personal stories that help schoolchildren to acquire knowledge on this matter.

**Study design:**

Pre and post-quasi-experimental study.

**Objective:**

To assess the increase in knowledge about road safety following a school-based road safety campaign.

**Methods:**

Two multiple-choice tests were given to each of the 8106 students taking part, who were 12–14 years old. Of the four possible answers, only one of them was correct. The first multiple-choice test was taken before the presentation and the second was taken one month later.

**Results:**

After assessing the answers, there was a change in the tendency of the number of correct before/after answers for the multiple-choice test, and the number of correct ones rose one month after the presentation. This increase is statistically significant (*p* < 0.01) and represents a national increase of 61% in the probability of correct answers, although this varies from 8% to 278% depending on the region.

**Conclusions:**

The assessment, involving over 8000 people, showed that there has been an improvement in road safety knowledge thanks to education provided by AESLEME’s instructors, and a statistically significant increase was obtained throughout Spain and different regions.

## Introduction

Road traffic accidents are a real pandemic and because they have been present in our societies for years, end up being given little attention as they are in the news every day.

The numbers are so great [https://www.who.int/features/factfiles/roadsafety/es/, https://www.who.int/roadsafety/decade_of_action/en/, https://ec.europa.eu/commission/presscorner/detail/es/qanda_20_1004, https://ec.europa.eu/eurostat/tgm/table.do?tab=table&init=1&language=en&pcode=sdg_11_40&plugin=1] [1] [https://www.epdata.es/datos/accidentes-trafico-datos-estadisticas/65/espana/106] [2] that we have become indifferent to them and they do not trigger a reaction in us. We must therefore take another look at this matter and imagine and visualise all the losses involved in it:

- More than 1.3 million people die each year worldwide. Road accidents are the leading cause of death among children and young people aged between 5 and 29.
- In the 27 member states of the European Union, about 23,000 people are killed—51 deaths per million inhabitants—and 1.5 million are injured.
- Spain is the sixth safest country in the EU with a mortality rate of 36 deaths per million inhabitants, just over 1000 deaths per year, and over 130,000 injuries each year, of which almost 10,000 require hospitalisation.

Lethality, or deaths/victims of road accidents, has also dropped and is around 1%, meaning it has decreased by three-quarters in two decades.

Prevention measures [3, 4] also exist but are not always implemented or followed: road improvement and signage, compliance with traffic regulations, and a system of fines in the event of infringements, in particular speeding, consumption of substances which reduce a driver’s ability as alcohol and other drugs, use of restraint systems and helmets for two-wheeled vehicles or improved vehicle design to make them safer for their occupants.

But often the key is road safety education offered at school to establish healthy habits when it comes to mobility [5].

Although the effectiveness of school campaigns is little studied or is not significatively related with road accidents prevention [6–11], is imperative to teach road safety from an early age as it helps to prevent the creation of attitudes that can become part of the chain of events that lead to road accidents, either as active agents—drivers, and sometimes passengers who accompany the driver, or pedestrians who jaywalk or walk in inappropriate places—or as passive agents, usually passengers and pedestrians.

Many countries continue to carry out these types of prevention campaigns, trying to modify behaviours and perceptions in schoolchildren yet without measuring in any way their effectiveness in preventing accidents. We believe that it is, at the very least, necessary to measure the knowledge gained with each campaign, although we do not know if, in the future, this will lead to fewer accidents involving the children who took part in our driver education classes. By using psychological tests, some research measures a possible change in attitudes that influence road traffic accidents, but these measurements are more complex to apply and cannot predict whether those who self-report a change in attitude suffer, or not, fewer accidents in the future [12].

AESLEME (Association for the Study of Spinal Cord Injuries) is a non-profit NGO, declared to contribute to the public good and accredited as a transparent NGO that follows ISO 39001 guidance on road safety and 9001 for quality, that has, for 30 years, dedicated itself to providing education on road safety and health to prevent road accidents. The Association’s activities also focus on psychological support for victims. In fact, most of AESLEME’s members have suffered a road accident, the consequences of which changed their lives, in a fraction of a second, forever. They are, therefore, very suitable instructors for disseminating prevention campaigns in places such as schools, universities, the armed forces and companies: they not only talk about risks, prevention measures, but also link this knowledge with their own life stories about the physical, psychological and social problems they face in their day-to-day lives. This captures the attention of audiences much more and becomes a powerful stimulus for them to modify their behaviour and adopt healthy habits when it comes to sharing the roads in a responsible way and contributing to safe mobility.

The Protection Motivation Theory [13] focuses on risk perception through perceived severity and vulnerability.

For this reason, the road education offered by AESLEME on knowledge about the causes, prevention and seriousness of road traffic accidents is combined with emotions and perceptions about the repercussions of said accidents [14, 15].

The objective of this paper is, to assess the increase in knowledge about road safety following a school-based road safety campaign.

## Methods

Among AESLEME’s instructors are healthcare workers and people with spinal cord injuries caused by road accidents; their presentations, which last an hour to one and a half hours, are interesting and are adapted to schoolchildren of different ages. The main strong point of each presentation is interaction with the audience, explaining not only the epidemiology of road accidents, the risks, and possible irreversible injuries that the instructors suffered, paraplegia or quadriplegia, but also experiences about their new reality. These are testimonies with which schoolchildren empathise and that motivate them to learn about what they have heard [14, 15].

To assess the schoolchildren’s knowledge acquisition of the basic concepts for the prevention of these kinds of accidents, AESLEME collaborated with several schools and high schools by giving presentations there. Prior permission, signed by the schools and by the parents, had to be drawn up and agreed upon. This quasi-experimental study, involving a pre and post-presentation, was conducted with a convenience sample that was based on accessibility to the population and willingness to participate in our investigation.

Two multiple-choice tests approved by our Directorate-General for Traffic were given to each student; of the four possible answers, only one was considered correct.

They took the first multiple-choice test before the presentation, and the second one a month later. Both questionnaires, which were identical, were answered in the classroom and each took 10 minutes to fill in.

The multiple-choice test is shown in the appendix and includes all the information the students were asked to give: age, gender, school or high school, their year, city, region, and year.

The answers, from two different academic courses, were rated as correct or incorrect and entered into a database to compare the before/after answers in each region and in the country. It was thus possible to determine how many students did not answer any question correctly before the presentation, and how many answered in the same way a month after the presentation. The same comparison was made with the correct answers for all 12 questions—12 being the maximum possible score—both before and after the educational presentation on traffic accident prevention.

Since the answers to both questionnaires were anonymous, we decided to compare the average and the percentages of correct answers to the 12 questions asked both times, exploring the increase in these percentages by means of odds ratios. The purpose of this study was to provide some descriptive values based on the results obtained before and after the presentation, but we were also able to assess the statistical significance associated to the intervention based on the average number of correct answers and on the percentages of children who answered the questionnaire correctly on both occasions.

The stratified study was carried out using the StatCalc programme (Epi Info, CDC), which calculated the results by the study’s variables, their respective chi-squared values and the odds-ratios (OR) which calculated the 2 × 2 tables obtained for Spain and several regions, their statistical significance and the OR with their respective 95% confidence limits.

### Reporting summary

Further information on research design is available in the Nature Research Reporting Summary linked to this article.

## Results

This study only presents the results of 8106 students aged between 12 and 14 years of age that corresponds to year 6 of primary school, or year 1 and 2 of secondary school i.e., 8106 multiple-choice tests were taken both before and after the presentation. The number of students varies by region depending on different reasons, for example: Andalusia, 776 students; Aragon, 435; the Canary Islands, 2520; Castilla La Mancha, 198; Extremadura, 670, according to the time the speakers had, as well as the possibilities and willingness of the schools to conduct these surveys. The results do not vary significantly with regard to age and sex, so, they have been presented together, without stratifying these factors.

Table 1 shows the percentages of students who answered correctly, before and after the presentation, stratified in rows, by the number of correct answers, and, as can be seen, 5% of students answered all of them correctly, even before the start of the presentation. Each column indicates where the surveys were conducted, and groups them by region or for Spain as a whole.

**Table 1:** Percentage of students (in quartils) according to the number of correct answers (before/after the AESLEME´s Road Education), globally in Spain or by different regions.

	Quartil 1	Quartil 2	Quartil 3	Quartil 4
	1–3 Cor.Ans.	4–6 Cor.Ans.	7–9 Cor.Ans.	10–12 Cor.Ans
Spain (before)	28.57%	35.75%	23.48%	12.2%
Spain (after)	20.29%	24.76%	25.33%	29.62%
Andalusia (before)	6.45%	48.58%	40.72%	4.25%
Andalusia (after)	1.68%	6.57%	30.8%	60.95%
Aragon (before)	5.85%	52.98%	38.67%	2.5%
Aragon (after)	0%	11.47%	62.19%	26.34%
Canary Islands (before)	21.59%	35.71%	25.47%	17.23%
Canary Islands (after)	26.35%	29.36%	19.37%	24.92%
CastM-Madrid (before)	10.2%	31.6%	49.1%	9.1%
CastM-Madrid (after)	0%	0%	38.4%	61.6%
Extremadura (before)	0.96%	42.79%	51.67%	4.58%
Extremadura (after)	1.5%	9.84%	48.06%	40.6%
Basque Country (before)	47.43%	29.38%	9.4%	13.95%
Basque Country (after)	26.01%	30.95%	20.43%	22.61%

*Cor.Ans.* correct answers, *CastM-Madrid* Castilla la Mancha-Madrid.

**Fig. 1:**
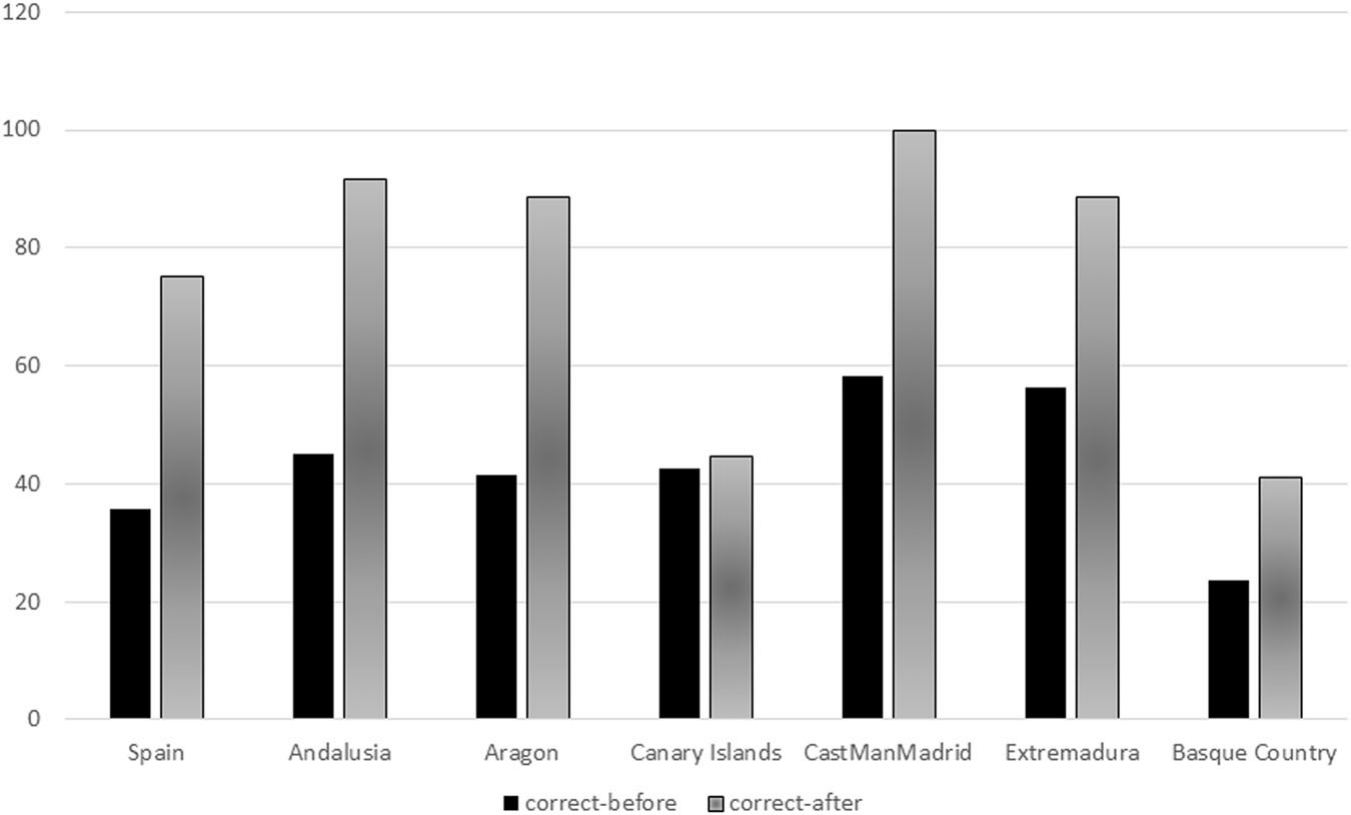
Percentage of students with 7–12 correct answers, before vs. after AESLEME’s Road Education.

As can be seen in Table 1 and Fig. 1, the global and regional data obtained for Spain.

The set of correct answers was calculated by region and for Spain as a whole. So, if 593 students each answered two questions correctly, we would have 1186 correct answers; or, if 473 students answered 12 questions correctly, we would have 5676 correct answers. The total number of possible correct answers would be the product of the number of students by the number of questions [7]. By knowing how many correct answers there were in one place, the number of incorrect answers—both before and after the presentation—can be assessed; this data can be used to draw up dichotomous tables with which the increase of knowledge can be calculated, both in Spain as well as in some specific regions by means of their odds ratio and their statistical significance.

Table 2 shows, the statistical significance, and the increase in correct answers after the presentation. As can be seen, this increase is statistically significant (*p* < 0.01 or more) and this means, globally, a 61% increase in correct answers after the presentation was given by the AESLEME speaker. The same thing was done in some regions: the increase of probability in correct answers went from only 8%, while others had very high scores, such as over 200%, which was double the number of correct answers after the presentation, all of which is a significant increase.

**Table 2:** Calculation of the increase in correct answers after the education on health given by AESLEME, for Spain as a whole and in some regions.

**Data for the whole of Spain:**			
	***Correct***	***Incorrect***	***Total***
*After*	56,529	40,743	97,272
*Before*	45,005	52,226	97,272
*Total*	101,534	93,010	194,554
Chi-squared = 2726 *p* < 0.00001; OR: 1.61; 95% CI :1.58 to 1.64			
Global increase in correct answers: 61% with 95% CI: 53% to 64%			
Andalusia:			
	***Correct***	***Incorrect***	***Total***
*After*	7499	1813	9312
*Before*	4862	4450	9312
*Total*	12,361	6263	18,624
Chi-squared = 672 *p* < 0.00001; OR: 3.78; 95% CI: 3.54 to 4.04			
Global increase in correct answers: 278% with 95% CI: 254% to 304%			
Aragon:			
	***Correct***	***Incorrect***	***Total***
*After*	3475	1745	5220
*Before*	2672	2548	5220
*Total*	6047	4393	10,440
Chi-squared = 255 *p* < 0.00001; OR: 1.89; 95 CI: 1.75 to 2.05			
The global increase in correct answers: 89% with 95% CI: 75% to 105%			
Canary Islands:			
	***Correct***	***Incorrect***	***Total***
*After*	15,900	14,343	30,240
*Before*	15,282	14,958	30,240
*Total*	31,182	29,298	60,480
Chi-squared = 25 *p* < 0.00001; OR: 1.08; 95% CI: 1.05 to 1.12			
Global increase in correct answers: 8% with 95% CI: 5% to 12%			

Castilla la Mancha and Madrid:			
	***Correct***	***Incorrect***	***Total***
*After*	1956	420	2376
*Before*	1371	1005	2376
*Total*	3327	1425	4752
Chi-squared= 343 *p* < 0.00001; OR: 3.41; 95% CI: 2.99 to 3.9.			
Global increase in correct answers: 241% with 95% CI: 199% to 290%			
Extremadura:			
	***Correct***	***Incorrect***	***Total***
*After*	5973	2067	8040
*Before*	4510	3530	8040
*Total*	10,483	5597	16,080
Chi squared= 586 *p* < 0.00001; OR: 2.26; 95% CI: 2.11 to 2.42			
Global increase in correct answers: 126% with 95% CI: 111% to 142%			
Basque Country:			
	***Correct***	***Incorrect***	***Total***
*After*	21,727	20,357	42,084
*Before*	16,312	25,772	42,084
*Total*	38,039	46,129	84,168
Chi-squared = 1406 *p* < 0.00001; OR: 1.68; 95% CI: 1.64 to 1.73			
Global increase in correct answers: 68% with 95% CI: 64% to 73%			

*OR* odds ratio, *95% CI* 95% Confidence Intervals.

## Discussion

Most countries have also implemented road safety campaigns. Different strategies have been used from very coercive ones, using deterrents or fining offenders, to more liberal ones based on raising awareness so that people adapt precautionary principles to changing day-to-day traffic situations, instead of just surveillance and punishment; these are not always effective, and also lead to behaviour that tries to thwart the effect of the surveillance as radar detectors or using computer programmes to warn other drivers that the police are at a particular junction, while a tendency to travel without adopting proper prevention strategies, and with it the risk of traffic accidents, continues.

A number of different campaigns to prevent road traffic accidents have been implemented by most countries using a variety of methods, intensity of application and duration. For example, the Centre for Road Safety in New South Wales has created campaigns, such as *Towards Zero*, *Saving Lives on Country Roads* or *Ride to Live*. In Spain, the Directorate-General for Traffic has designed campaigns, such as *The Glass Man*, *Top Holiday*, *Distractions*, but in AESLEME´s campaigns a differential element has been introduced: knowledge is accompanied by testimonies and the view of someone who has suffered this kind of accident, which provides credibility and great conviction to everything that is said. In addition, teaching methods have greatly improved since the first presentations given 30 years ago, and virtual reality together with enhanced audiovisual mediums, allows for a better adaptation to different audiences. Our statistical results were expected as our initial premise was about conveying knowledge in addition to transmitting emotion, and this knowledge was linked to the number of correct answers.

In each scholar class, the ratio of boys to girls tend to 1 (same number of both sexes) and the age range chosen for this study is small (12 to 14 years). It is therefore logical that these two variables make no difference on whether they have more or less knowledge on the road accidents prevention. In other studies, which includes a broader age range, the end up influencing the knowledge acquired during a training, but not sex, which remains insignificant.

The study was easy to conduct, but risky in its objectives:

-Easy, because each teenager was not given an identification number, which could be lost, changed or forgotten, since they did not know which specific day the second part of the study was going to be conducted. This prevented an individual comparative study of the ‘before/after’ responses, but not a joint study of the whole class, which also demonstrated the effect of the didactic presentation given.
-Risky, since, if the presentation had not left a sufficiently powerful memory, the ‘before/after’ result would have tended towards a ‘null effect’. However, if the effect was positive after a month, it would be a sign of integration of this knowledge in adolescents, which could lead to behaviour modification towards greater road safety.

As can be seen by the statistics, this didactic approach has proven to be effective for acquiring knowledge in just 1½ hours, in Spain as a whole and in the regions where the study was conducted with similar multiple-choice tests taken before and then one month later. The results varied in their degree of effectiveness, as we have seen, but they always point in the same direction—in other words, the consistency of the effect—despite being conducted by different people and in very different places; this proves that the method chosen is reliable and makes this average increase in knowledge of 61% very valuable, compared with those who participated in the survey before starting the road safety education activity. These good results support the Protection Motivation Theory [13], which focuses on risk perception through perceived severity and vulnerability.

Limitations of the study: the number of before/month-after answers of each of the schoolchildren have not been studied due to the design of the survey as it ensures anonymity. This also complicated the type of statistical studies that demonstrate the effect. However, despite only having this overall assessment by school year, school, city and region, a huge effect was appreciated after one month, even without any further education, which is most likely due to the great emotional and educational impact contained in the presentation given by AESLEME’s instructors.

As it depended on the willingness and facilities provided by each school for the instructors to return the following month and take the second survey, it was not conducted in every region in Spain. We, therefore, believe that there was no response bias in the schools that collaborated, yet the increase in knowledge was significant in all of them.

Finally, it is impossible to know if what has been examined by this paper will to provide road accident prevention. We cannot affirm that this knowledge translated into fewer road accidents. This would require a huge cohort study, that is, a follow-up of a great number of children having received this driver´s education and a control group that did not receive such education. Furthermore, the follow-up would have to last for many years. But there are studies [12, 16–22] that demonstrate a positive association between knowledge of traffic rules, better risk perception and future safer road behaviours, provided that the programmes are highly educational and adapted to the profile of the students. In addition, they should be complemented with excursions to safe urban circuits monitored by teachers and urban police officers in order to further cement the theory with practical classes [22].

It must also be said that not all researchers [22, 23] agree with this correlation between knowledge and preventive attitudes to reduce road accidents, since there are many other factors involved in this causal chain, such as the example and attitudes of parents and friends, which can antagonise, or synergise, preventive attitudes derived from any traffic accident prevention programme [24, 25].

However, we believe, as do other authors [12], that school environments are particularly conducive for laying the foundations for the acquisition of the correct attitudes for preventing traffic accidents. This is because they act on very large populations at a key moment of their lives, in which many of their attitudes to life are shaped; this is even more true if the educational programme includes a presentation as impactful as ours, the effects of which remain a month after being carried out.

## Main conclusions

-Increased knowledge on road safety was assessed in over 8000 people one month after presentations offered by AESLEME’s instructors, with the attainment of an average in Spain of 61% in the probability of correct answers, which is statistically important.
-This increase was also important with regard to the different regions studied, from 8% to more than 200%.

## Supplementary information

Reporting Summary

## Data Availability

The datasets, analysed during the concurrency study, are available from AESLEME.
